# Synthesis and acaricidal activity against *Varroa destructor* of α- and γ-costic acid dimers

**DOI:** 10.3762/bjoc.22.87

**Published:** 2026-07-21

**Authors:** Alessandro Santarsiere, Ernesto Santoro, Maria Letizia Ciavatta, Marianna Carbone, Sonia Ganassi, Cosimo Tedino, Antonio De Cristofaro, Antonio Evidente, Stefano Superchi

**Affiliations:** 1 Department of Basic and Applied Sciences, Universty of Basilicata, Via dell’Ateneo Lucano 10, I-85100 Potenza, Italy; 2 National Research Council, Institute of Biomolecular Chemistry, Via Campi Flegrei 34, I-80078 Pozzuoli (NA), Italy; 3 Department of Agricultural, Environmental and Food Sciences, University of Molise, Via F. De Sanctis snc, I-86100 Campobasso, Italy

**Keywords:** acaricidal activity, α-costic acid, γ-costic acid, diesters, *Varroa destructor*

## Abstract

α-Costic acid is a natural sesquiterpene possessing diverse biological activities. Among these, the most promising property for practical application is its acaricidal activity against *Varroa destructor*, considered the most important and dangerous parasite of the honeybee (*Apis mellifera* L.). Infestations of *V. destructor* can decimate bee populations in just a few years, resulting in substantial environmental and economic losses. This study reports the synthesis of α- and γ-costic acid diesters with ethylene glycol and evaluates their acaricidal efficacy against *V. destructor* in comparison with their parent acids. Although the resulting dimers were approximately 50% less active than the parent costic acids, they exhibited higher potency than the previously reported α-costic acid methyl ester. Among all, γ-costic acid, whose acaricidal activity is reported here for the first time, shows the highest activity with mortality higher than 90%, thus emerging as the most promising candidate for the development of effective and environmentally friendly strategies for the control of *V. destructor*. Furthermore, it may hold potential for the biocontrol of other mite pests affecting economically important crops such as legumes and cereals, which are currently associated with significant yield losses and extensive reliance on synthetic pesticides.

## Introduction

**Figure 1:**
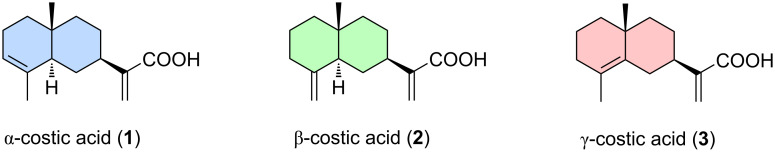
Structures of costic acid isomers.

11(13)-Eudesman-12-oic acids are sesquiterpene plant metabolites widely distributed in nature and produced by different wild plant genera [1]. In particular, α-costic acid (**1**), also known as isocostic acid, (Figure 1) was first isolated from aerial parts of *Dittrichia viscosa* (syn. *Inula viscosa*) [2] and *Dittrichia graveolens* (L.) Greuter (syn. *Inula graveolens*) [3], herbaceous perennial plants widespread throughout Mediterranean basin, and of *Cratystylis microphylla S*. collected in Australia [4]. The isomeric β-costic acid (**2**), also known as costic acid (Figure 1), was isolated from costus root oil [5] and later, together with the structurally related ilicic, viscic and viscosic acids, from *D. viscosa* collected in southern Turkey [6]. The third known structural isomer of **1**, γ-costic acid (**3**) (Figure 1), was obtained through chemical transformation of either ilicic acid [7] or of the parent compound **1** [8].

α-Costic acid (**1**) has shown various biological activities. Regarding its potential applications in the agro-food sector, it was found to inhibit *Cuscuta campestris* and stimulate *Orobanche crenata* seed germination [9]. Its mechanism of action in plant cells has been also recently investigated [10]. Probably, the most interesting property of compound **1** is its acaricidal activity against *Varroa destructor* that is considered the most important and dangerous parasite of the honeybee (*Apis mellifera* L.). *V. destructor* is able to completely suppress infested honeybee populations within a few years [11–12] with severe economic consequences [13]. A significant acaricidal effect was observed when the parasitic mite was exposed to α-costic acid (**1**) while no adverse effects were observed in honeybees [14]. Furthermore, **1** has been shown to possess acaricidal activity against the cattle ectoparasitic tick, *Rhipicephalus (Boophilus) annulatus* [15], fungicidal activity against both *Aspergillus niger* and *Penicillium roqueforti* [16], as well as protectant activity for chickpea seeds against the cowpea seed beetle *Callosobruchus maculatus* (Coleoptera: Chrysomelidae) [17]. Although comparable acaricidal activity against *V. destructor* has been reported for β-costic acid (**2**) [18] this specific activity has never been reported for γ-costic acid (**3**), for which only antifeedant [19] and antibacterial activities [8] have been demonstrated.

Recently, some of us reported that the synthesized dimeric esters of both polygodial and ophiobolin A with ethylene glycol show enhanced anticancer activity with respect to their parent compounds shifting potencies from two-digit to single-digit micromolar levels against apoptosis-resistant cancer cells [20]. Similar increases in anticancer activity have been also reported for dimers of other natural bioactive compounds [20]. Consequently, it was of interest to investigate whether ethylene glycol ester dimers of α-costic acid (**1**) or γ-costic acid (**3**) would enhance their bioactivity as well. Accordingly, we herein report the synthesis, chemical characterization, and acaricidal activity against *V. destructor* of α,α- and γ,γ-costic acid homodimers and α,γ-costic acid heterodimer esters in comparison with their parent monomeric acids.

## Results and Discussion

**Scheme 1:**
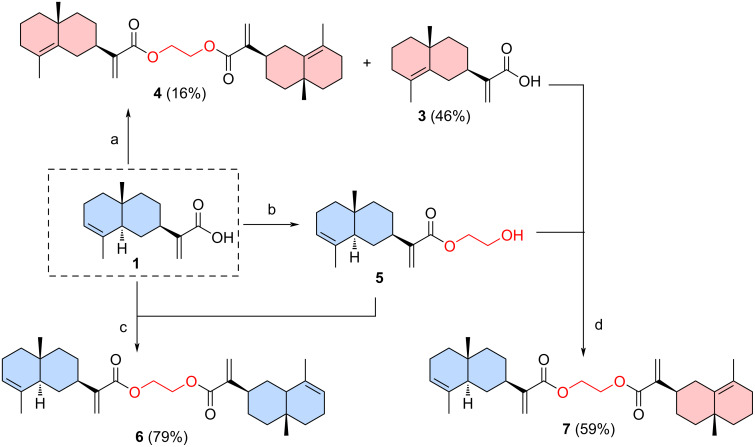
Synthesis of costic acids derivatives. Reaction conditions: (a) ethylene glycol (0.5 equiv), *p*TsOH (0.1 equiv), toluene (2.0 mL/mmol), 100 °C, 4 h; (b) DMAP (0.1 equiv), DCC (1.4 equiv), CH_2_Cl_2_ (6.0 mL/mmol), ethylene glycol (1.0 equiv), 0 °C to rt, overnight; (c) DMAP (0.1 equiv), DCC (1.4 equiv), CH_2_Cl_2_ (6.0 mL/mmol), **1** (1.0 equiv), 0 °C to rt, overnight; (d) DMAP (0.1 equiv), DCC (1.4 equiv), CH_2_Cl_2_ (6.0 mL/mmol), **3** (1.0 equiv), 0 °C to rt, overnight.

Given the significant economic and ecological impact of *V. destructor*, considerable efforts have been devoted to the development of acaricides to control this ectoparasite [21]. However, the use of synthetic pesticides poses risks to both human and animal health, as their residues have been detected in honey samples [18]. Moreover, numerous studies have demonstrated that these compounds can exert both lethal and sublethal effects on honeybees and may accumulate in bee-derived products [22–24]. In addition, the efficacy of both synthetic and natural acaricides, such as formic and oxalic acids [25], has declined due to the development of resistance in *V. destructor* populations [26]. Consequently, there is a growing interest in alternative, efficient, and environmentally friendly control strategies based on natural compounds. To date, both α-costic acid (**1**) [14] and β-costic acid (**2**) [18] have shown promising results against *V. destructor*, whereas no studies have reported the acaricidal properties of γ-costic acid (**3**). Therefore, we deemed it worthwhile to investigate the acaricidal activity of γ-costic acid (**3**) against *V. destructor*. Furthermore, since dimerization has, in some cases, been shown to enhance the biological activity of various natural compounds [20], we also aimed to compare the acaricidal activities of costic acids **1** and **3** with those of their corresponding homo- and heterodimers **4**, **6**, and **7** (Scheme 1).

Accordingly, the formation of dimer of **6** with ethylene glycol was first attempted under classical acid-catalyzed Fischer esterification conditions, in the presence of *p*-toluenesulfonic acid (*p*TsOH) (Scheme 1). However, these conditions led to isomerization of **1** into γ-costic acid (**3**) which was isolated together with its dimeric ester **4**. On the contrary, Steglich esterification, achieved under basic catalysis by treating α-costic acid (**1**) with 1.0 equiv of ethylene glycol in the presence of dicyclohexylcarbodiimide (DCC) and a catalytic amount of *N*,*N*-dimethylaminopyridine (DMAP), provided the monoester **5** and no trace of isomerization products. Finally, both the α-costic acid homodimer **6** and the α/γ-costic acid heterodimer **7** were obtained by Steglich esterification of **5** with α-costic acid (**1**) and γ-costic acid (**3**), respectively (Scheme 1).

**Figure 2:**
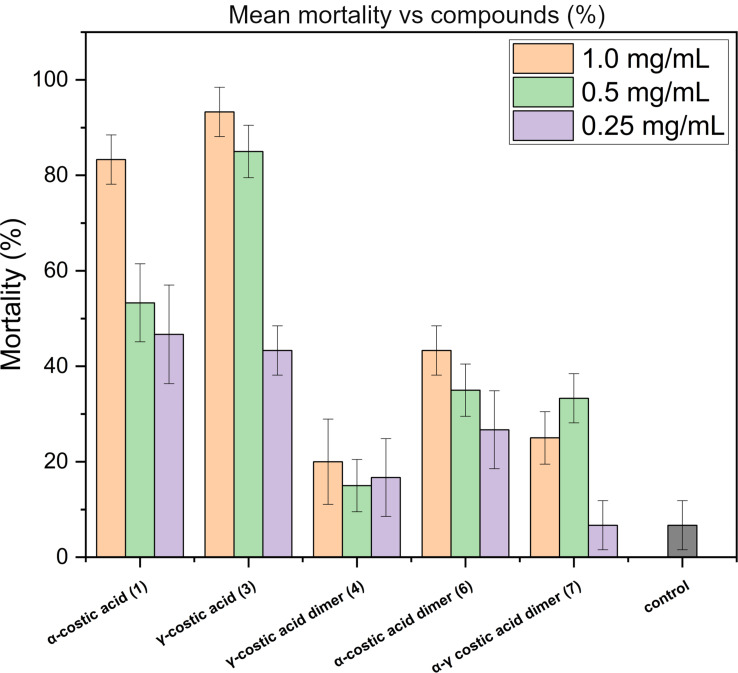
Mean mortality vs compounds (%).

The acaricidal activity of the costic acid derivatives, comprising the two monomers **1**, **3**, and the three dimers **4**, **6**, **7**, was evaluated in vitro against *V. destructor* at three concentrations (0.25, 0.5 and 1.0 mg/mL) (Table 1 and Figure 2). The efficacy of the compounds was determined based on the mortality observed after 24 hours and one-way ANOVA performed on the mean mortality values revealed significant differences across the treatments (F_(18;95)_ = 98.614; *P* < 0.001) (Table 1 and Figure 2).

**Table 1:** Effect of five costic acid derivatives on *V. destructor.* Different letters indicate statistically significant differences (Tukey test, *P* < 0.05).

Compounds	Mean mortality ± SD 1.0 mg/mL	Mean mortality ± SD 0.5 mg/mL	Mean mortality ± SD 0.25 mg/mL

α-costic acid (**1**)	8.33 ± 0.516^a^	5.33 ± 0.816^b^	4.67 ± 1.033^bc^
γ-costic acid (**3**)	9.33 ± 0.516^a^	8.50 ± 0.548^a^	4.33 ± 0.516^bcd^
γ-costic acid dimer (**4**)	2.00 ± 0.894^efg^	1.50 ± 0.548^fg^	1.67 ± 0.816^fg^
α-costic acid dimer (**6**)	4.33 ± 0.516^bcd^	3.50 ± 0.548^cde^	2.67 ± 0.816^ef^
α-γ costic acid dimer (**7**)	2.50 ± 0.548^ef^	3.33 ± 0.516^cde^	0.67 ± 0.516^g^
control	0.67 ± 0.516^g^	0.67 ± 0.516^g^	0.67 ± 0.516^g^

Surprisingly, γ-costic acid (**3**) exhibited the highest acaricidal efficacy, reaching a mortality rate of 93.3% at 1.0 mg/mL. This potency remained remarkably high at 0.5 mg/mL (85.0%) and still significant (43.3%) even at the lowest dose. In comparison, the parent α-costic acid (**1**) showed a lower mortality of 83.3% at 1.0 mg/mL, decreasing to 53.3% at 0.5 mg/mL and 46.7% at 0.25 mg/mL, still displaying a strong dose-dependent activity. Overall, the dimers **4**, **6**, and **7** showed significantly lower efficacy than the corresponding monomers and marked variability in results (Table 1 and Figure 2). The α-costic acid homodimer **6** was the most active of three, with a maximum mortality of 43.3% at 1.0 mg/mL and a dose-dependent trend, although with values that were always much lower than those of the monomer. The γ-costic acid homodimer **4** showed stable but lower mortality (15–20%) at all doses, while the α,γ-heterodimer **7** showed comparable activity at the medium and high concentration but no activity at the lowest concentration, revealing 6.7% mortality as the control. In summary, only dimer **6** exceeded 43% mortality indicating reduction in biological activity compared to the monomeric forms. In the negative control (2% DMSO + 0.9% NaCl), the average mortality was 6.7%, confirming that the solvent vehicle did not significantly affect mortality.

These results show that the acaricidal activity of costic acid derivatives is highly structure-specific and dose-dependent, with γ-costic acid (**3**), the acaricidal activity of which was never reported before, emerging as the most promising candidate for the development of effective and environmentally friendly strategies for the control of *V. destructor*. To highlight the potential of the costic acid derivatives in *V. destructor* control, their activity has been also compared with that reported in the literature for compound **2** and for widely employed natural acaricides as formic acid (**8**) and oxalic acid (**9**) (Table 2). Whereas very variable acaricidal activity has been reported, ranging from 39.8% [27] to 92% [28] mite mortality, for formic acid (**8**), a recent formulation study showed 93.1% mortality [29] for oxalic acid (**9**). This activity is comparable with that obtained in our study with γ-costic acid (**3**), supporting one more time the potential of compound **3** as a valuable alternative to existing synthetic and natural acaricides for *V. destructor* control.

**Table 2:** Comparison of mean mortality with known soft acaricides.

Compounds	Mean mortality	Reference

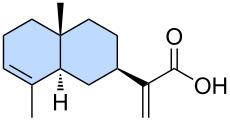 α-costic acid (**1**)	83%	this work
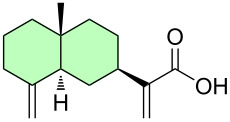 β-costic acid (**2**)	80–100%	[30]
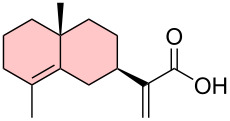 γ-costic acid (**3**)	93%	this work
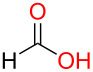 formic acid (**8**)	39.8–92%	[27–28]
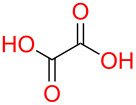 oxalic acid (**9**)	up to 93%	[29]
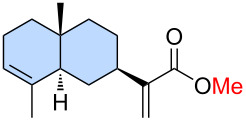 α-costic acid methyl ester (**10**)	10%	[14]

**Figure 3:**
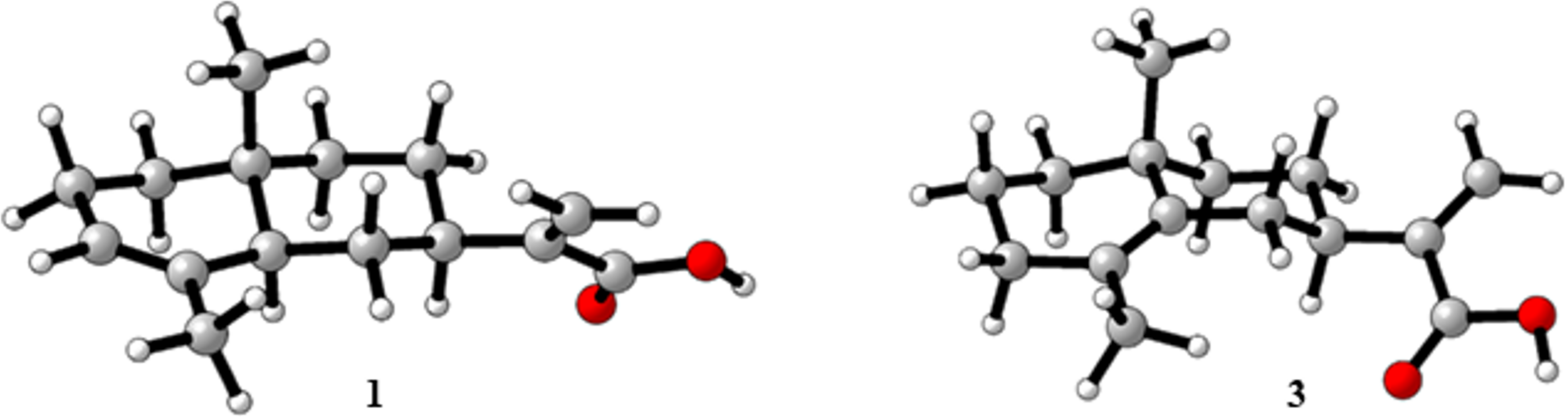
Computed main conformers (DFT/B3LYP/def2-TZVP/gas phase) of compound **1** (left picture) and compound **3** (right picture).

The structural features that are mostly involved in the acaricidal activity of costic acid derivatives are both the presence of a free carboxylic acid group and of an α,β-unsaturated carboxyl moiety. In fact, moving from α-costic acid (**1**) to its methyl ester **10** [14] a marked decrease in activity from 83% to 10% is observed and, as reported above, the transformation of costic acids **1** and **3** to diesters **4**, **6**, **7** led to a decrease in acaricidal activity from 80–90% to 20–40%. The same activity decrease was displayed by costol, i.e. the allylic alcohol derived by reduction of the carboxyl moiety of **2** [30], where both the carboxyl and the α,β-unsaturated carboxyl moieties are lacking. On the contrary, the endo or exocylic position of the double bond on the A cycle of the costic acids does not exert a major effect on the acaricidal activity, which is of the same order of magnitude for the three isomers **1**–**3**. The 10% activity difference between compounds **1** and **3** can be due to the change in the spatial orientation of the bicyclic system and its associated substituents determined by the different ring junction in the two compounds (Figure 3), probably affecting the interaction with the biological receptors.

## Conclusion

This study reports the synthesis of α- and γ-costic acid diesters with ethylene glycol and evaluates their acaricidal activity, alongside that of the corresponding monomeric acids, against the ectoparasite *V. destructor*. Overall, the dimeric derivatives exhibited approximately 50% lower activity than the parent costic acids **1** and **3**. This reduction in efficacy may be attributed to incomplete or inefficient metabolic cleavage within target cells, resulting in only partial release of the active monomeric forms. Among the compounds tested, γ-costic acid (**3**), whose acaricidal activity had not been previously reported, demonstrated the highest potency, achieving 93% mortality. This activity surpasses that of the known α-costic acid (**1**) and β-costic acid (**2**). The superior performance of derivative **3** may be explained by its distinct conformational orientation (Figure 3), which likely enhances its interaction with biological targets.

These findings highlight γ-costic acid (**3**) as a promising candidate for the development of effective and environmentally sustainable strategies to control *V. destructor*. Furthermore, taking into account that previous studies [19] have shown no cytotoxic activity of γ-costic acid (**3**) to mammalian cells, this compound may hold potential for the biocontrol of other mite pests affecting economically important crops such as legumes and cereals [31], currently associated with significant yield losses and extensive reliance on synthetic pesticides [32].

## Experimental

### General

All reagents were supplied by Sigma-Aldrich (Milan, Italy) and TCI (Tokyo, Japan) and were used without further purification unless otherwise stated. Flash chromatography was performed using 60–200 mesh silica gel. ^1^H NMR and ^13^C NMR spectra were recorded on Varian (Palo Alto, CA, USA) 400 MHz and 500 MHz and 100 MHz and 125 MHz spectrometers, respectively, at ambient temperature in CDCl_3_, unless otherwise noted, using the same solvent as internal standard at δ (ppm) 7.26 and 77.0. Chemical shifts are given in δ (ppm). Data for ^1^H NMR are reported as follows: chemical shift, multiplicity (s = singlet, brs = broad singlet, d = doublet, brd = broad doublet, brdd = broad double doublet, m = multiplet), coupling constants (in hertz) and integration. Low and high-resolution mass spectra (LR- and HRESIMS) were acquired on a Q-Exactive hybrid quadrupole-orbitrap mass spectrometer (Thermo Scientific, San Jose, CA, USA). Analytical and preparative thin-layer chromatography (TLC) was carried out on silica gel, Kieselgel 60, F254, 0.25 and 0.5 mm plates (Merck, Darmstadt, Germany), respectively. Column chromatography was performed on a silica gel column, Kieselgel 60, 0.063–0.200 mm (Merck).

### Synthesis of γ-costic acid (**3**) and γ-costic acid diester (**4**)

α-Costic acid (**1**, 1.0 equiv), ethylene glycol (0.5 equiv) and *p*-toluenesulfonic acid (0.1 equiv) were dissolved in anhydrous toluene (2.0 mL/mmol), the reaction mixture was heated at 100 °C and stirred for 4 hours. The solvent was then removed under reduced pressure. The crude residue was purified by column chromatography (eluent: petroleum ether/EtOAc 8:2) to afford the γ-costic acid diester (**4**) (16%) and γ-costic acid (**3**) (46%). Compound **3**: ^1^H NMR (400 MHz) δ 6.33 (s, 1H), 5.69 (s, 1H), 2.66 (d, *J* = 13.6 Hz, 1H), 2.37 (d, *J* = 16.4 Hz, 1H), 2.01–1.85 (m, 2H), 1.75 (m, 1H), 1.69 (m, 1H), 1.64–1.46 (m, 7H), 1.42–1.23 (m, 3H), 1.05 (s, 3H). Compound **4**: ^1^H NMR (400 MHz) δ 6.19 (s, 2H), 5.58 (s, 2H), 4.42 (s, 4H), 2.62 (m, 2H), 2.38 (m, 2H), 1.91 (m, 4H), 1.81 (m, 2H), 1.63 (m, 2H), 1.59 (s, 6H), 1.55 (m, 6H), 1.43 (m, 2H), 1.36–1.26 (m, 4H), 1.25 (m, 2H), 1.04 (s, 6H); ^13^C NMR (100 MHz, CDCl_3_) δ 167.18, 145.55, 134.36, 125.37, 123.23, 62.45, 42.25, 40.72, 40.30, 34.57, 33.26, 31.43, 28.22, 24.75, 19.49, 19.20; HRESIMS (*m/z*): [M + Na]^+^ calcd for C_32_H_46_O_4_Na, 517.3294; found, 517.3299.

### Synthesis of α-costic acid monoester (**5**)

α-Costic acid (**1**, 1.0 equiv) was dissolved in anhydrous CH_2_Cl_2_ (6.0 mL/mmol) and cooled to 0 °C, then DMAP (0.1 equiv) and DCC (1.4 equiv) were successively added. After stirring the solution for 5 minutes at 0 °C, ethylene glycol (1.0 equiv) was added to the resulting whitish suspension and the reaction mixture was allowed to warm to room temperature overnight. The white precipitate was removed by filtration and rinsed with CH_2_Cl_2_. The solvent was then removed under reduced pressure. ^1^H NMR analysis of crude showed the presence of compound **5** with minor impurities coming from unreacted **1**. Compound **5** was employed for the subsequent transformations without further purification. ^1^H NMR (500 MHz) δ 6.19 (s, 1H), 5.59 (s, 1H), 5.30 (brs, 1H), 4.30 (m, 2H), 3.88 (m, 2H), 2.51 (m, 1H), 2.07 (m, 1H), 1.93–2.03 (m, 2H), 1.82 (brd, *J* = 13.0 Hz, 1H), 1.62 (m, 1H), 1.58 (s, 3H), 1.55 (m, 1H), 1.45 (m, 1H), 1.36–1.32 (m, 2H), 1.22–1.28 (m, 2H), 0.80 (s, 3H).

### Synthesis of α-costic diester (**6**)

α-Costic acid (**1**, 1.0 equiv) was dissolved in anhydrous CH_2_Cl_2_ (6.0 mL/mmol) and cooled to 0 °C, then DMAP (0.1 equiv) and DCC (1.4 equiv) were successively added. After stirring the solution for 5 minutes at 0 °C, a solution of monoester **5** (1.0 equiv) in CH_2_Cl_2_ (4 mL/mmol) was added to the resulting whitish suspension. The reaction mixture was allowed to warm to room temperature and stirred overnight. The white precipitate was removed by filtration and rinsed with CH_2_Cl_2_. The solvent was then removed under reduced pressure. The crude residue was purified by column chromatography (eluent: petroleum ether/EtOAc 8:2) to afford α-costic acid diester **6** (79%). ^1^H NMR (500 MHz) δ 6.17 (s, 2H), 5.58 (s, 2H), 5.32 (brs, 2H), 4.42 (s, 4H), 2.51 (m, 2H), 2.09 (m, 2H), 1.93–2.03 (m, 4H), 1.82 (brd, *J* = 12.7 Hz, 2H), 1.63 (m, 2H), 1.58 (s, 6H), 1.55 (brdd, *J’* = 12.94 Hz, *J’’* = 3.8 Hz, 2H), 1.46 (dt, *J’* = 13.08 Hz, *J’’* = 3.63 Hz, 2H), 1.38–1.34 (m, 4H), 1.21–1.28 (m, 4H), 0.81 (s, 6H); ^13^C NMR (125 MHz) δ 166.96, 145.50, 134.63, 123.16, 120.95, 62.18, 46.73, 40.48, 40.02, 37.69, 32.15, 29.18, 27.28, 22.83, 21.01, 15.53; HRESIMS (*m/z*): [M + Na]^+^ calcd for C_32_H_46_O_4_Na, 517.3294; found, 517.3297.

### Synthesis of α-costic-γ-costic acid mixed ester (**7**)

γ-Costic acid (**3**, 1.0 equiv) was dissolved in anhydrous CH_2_Cl_2_ (6.0 mL/mmol) and cooled to 0° C, then DMAP (0.1 equiv) and DCC (1.4 equiv) were successively added. After stirring the solution for 5 minutes at 0 °C, a solution of α-costic monoester **5** (1.0 equiv) in CH_2_Cl_2_ (4 mL/mmol) was added to the resulting whitish suspension and the reaction mixture was allowed to warm to room temperature overnight. The white precipitate was removed by filtration and rinsed with CH_2_Cl_2_. The solvent was then removed under reduced pressure. The crude was purified by column chromatography (eluent: petroleum ether/AcOEt 8:2) to afford the desired product **7** (59%). ^1^H NMR (500 MHz, CDCl_3_) δ 6.19 (s, 1H), 6.17 (s, 1H), 5.58 (s, 2H), 5.31 (brs, 1H), 4.42 (s, 4H), 2.63 (m, 1H), 2.51 (m, 1H), 2.38 (s, 1H), 2.09 (m, 1H), 1.91–2.03 (m, 4H), 1.84–1.90 (m, 2H), 1.82 (m, 2H), 1.63 (m, 2H), 1.59 (s, 6H), 1.55 (m, 4H), 1.41–1.46 (m, 2H), 1.38–1.25 (m, 4H), 1.26–1.19 (m, 3H), 1.04 (s, 3H), 0.81 (s, 3H); ^13^C NMR (125 MHz, CDCl_3_) δ 167.50, 145.95, 145.78, 135.16, 134.60, 125.62, 123.69, 123.43, 121.40, 62.72, 62.66, 47.20, 42.49, 40.95, 40.53, 38.18, 34.81, 33.50, 32.65, 31.63, 29.66, 28.44, 27.79, 24.96, 23.33, 21.55, 19.74, 19.44, 16.06, 16.02; HRESIMS (*m/z*): [M + Na]^+^ calcd for C_32_H_46_O_4_Na, 517.3294; found, 517.3298.

### Plant material, extraction, and purification of α-costic acid

Whole aerial parts of *Dittrichia viscosa* plants were freshly collected in Algeria from naturally occurring populations. A voucher specimen was deposited at the herbarium of the Ecole National Supérieure Agronomique in Algiers. After harvesting, leaves were detached from the stems and dried in a ventilated oven at 50 °C for two days. The dried plant (400 g) was extracted as previously described in detail and the methylene chloride extract purified by different steps of column chromatography and TLC to afford α-costic acid (**1**, 455 mg, 1.14 g/kg) as a pale-yellow oil [9].

### General procedure for mortality test

Adult females of *V. destructor* were obtained from naturally infested and untreated Italian honeybee (*Apis mellifera ligustica*) colonies maintained at the experimental apiary of the University of Molise, located in Vinchiaturo (Campobasso, Italy): 41°52'417"N; 14°622'218"E. Mites were collected from sealed worker brood cells, sampled a few hours before adult bee emergence. Brood comb sections, containing capped worker pupae at a late developmental stage, were transferred to the laboratory and maintained in an incubator at 34.5 ± 0.5 °C and 65 ± 5% relative humidity, according to conditions commonly adopted for laboratory studies on *V. destructor*, and honeybee brood development [33]. Newly emerged adult female mites were recovered from brood cells as bee emergence occurred. Only active and healthy mites showing normal mobility and no visible physical damage were selected for the experiments and used within a few hours after collection.

### Acaricidal activity

Mortality tests were conducted under laboratory conditions, as previously reported [14], with slight modifications. The tests were conducted in Petri dishes (diameter 3.5 cm) with a 4 cm diameter filter paper disc (Whatman 1) placed at the bottom and along the vertical side for 0.25 cm. For each replication, 200 microliters of tested solutions and control were used, and the mites were gently placed on the filter paper with a brush. The Petri dishes were sealed with Parafilm, incubated at 25 °C, and after 24 h of exposure, mite mortality was assessed by gently stimulating each mite with a thin brush. Mites showing no movement in response to stimulation were transferred to new Petri dish containing a dry filter paper disc to confirm the absence of vital signs. After 1 h, mites that were unable to move a distance equal to or greater than their body length following gentle stimulation with a thin brush were considered as dead [34].

The acaricidal activity of five costic acid derivatives **1**, **3**, **4**, **6**, and **7** was evaluated in vitro on *V. destructor* at three concentrations (0.25, 0.5 and 1.0 mg/mL), with 10 mites per replicate and six replicates per treatment. The negative control was 2% DMSO + 0.9% NaCl. The efficacy of the compounds was determined based on the mortality observed after 24 hours.

### Computations

Geometry optimizations and frequency calculations of compounds **1** and **3** were performed by density functional theory (DFT) computations at DFT/B3LYP/def2-TZVP/gas phase level of theory using the ORCA 6.0 package [35].

### Statistical analysis

Mean mortality data of *V. destructor*, in the presence of the five costic acid derivatives tested at different concentrations, were analyzed by One-Way ANOVA, and the differences between the mean values were determined by the use of multiple-comparison test (Tukey test, *P* < 0.05). Data were processed by statistical package for social sciences (SPSS), version 27.0 per Windows software (SPSS Inc., Chicago, IL).

### Biosafety approval

No specific biosecurity approval was required for the experimental work involving *Varroa destructor* according to the applicable national regulations. All experiments were nevertheless conducted in compliance with institutional biosafety and containment guidelines at Department of Agricultural, Environmental and Food Sciences, University of Molise (Campobasso, Italy).

## Supporting Information

File 1 NMR and HRESIMS spectra of compounds **4**, **6** and **7**.

## Data Availability

All data that supports the findings of this study is available in the published article and/or the supporting information of this article.
