# Supplementary material for: Synthesis and acaricidal activity against Varroa destructor of α- and γ-costic acid dimers
Source: Beilstein J Org Chem. 2026 Jul 21;22:1088–96. doi: 10.3762/bjoc.22.87 (PMC13402994; doi:10.3762/bjoc.22.87)
Supplement: File 1 — NMR and HRESIMS spectra of compounds 4, 6 and 7. [file Beilstein_J_Org_Chem-22-1088-s001.pdf]

BEILSTEIN JOURNAL OF ORGANIC CHEMISTRY

Supporting Information

for

Synthesis and acaricidal activity against *Varroa destructor* of α- and γ-costic acid dimers

Alessandro Santarsiere, Ernesto Santoro, Maria Letizia Ciavatta, Marianna Carbone, Sonia Ganassi, Cosimo Tedino, Antonio De Cristofaro, Antonio Evidente and Stefano Superchi

Beilstein J. Org. Chem. **2026,** *22,* 1088–1096. doi:10.3762/bjoc.22.87

NMR and HRESIMS spectra of compounds 4, 6 and 7

License and Terms: This is a supporting information file under the terms of the Creative Commons Attribution License (https://creativecommons.org/licenses/by/4.0). Please note that the reuse, redistribution and reproduction in particular requires that the author(s) and source are credited and that individual graphics may be subject to special legal provisions.
The license is subject to the *Beilstein Journal of Organic Chemistry* terms and conditions: (https://www.beilstein-journals.org/bjoc/terms)

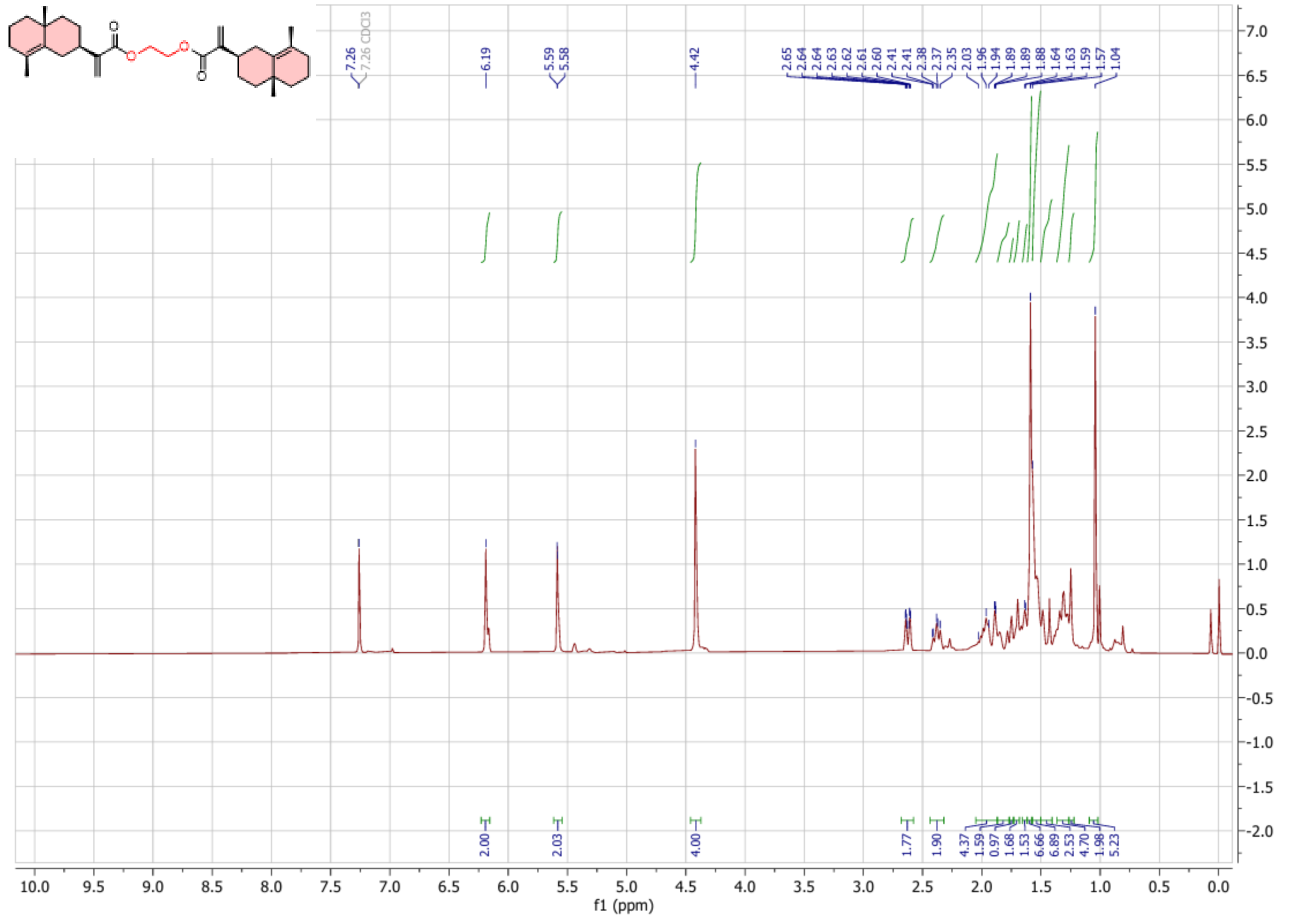

Figure S1. ^{1}H NMR (400 MHz, $CDCl_3$) spectrum of compound **4**.

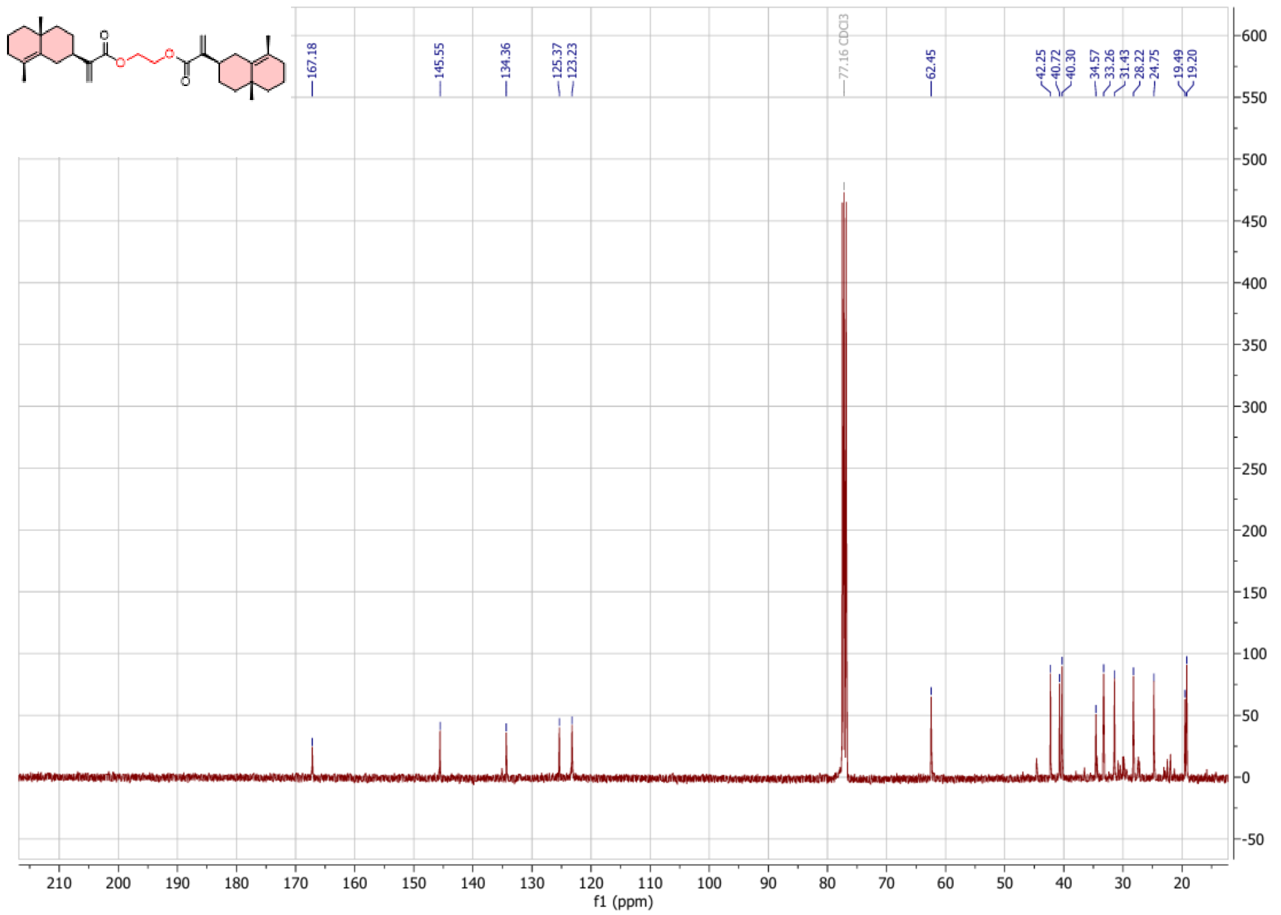

Figure S2. ^{13}C NMR (100 MHz, $CDCl_3$) spectrum of compound **4**.

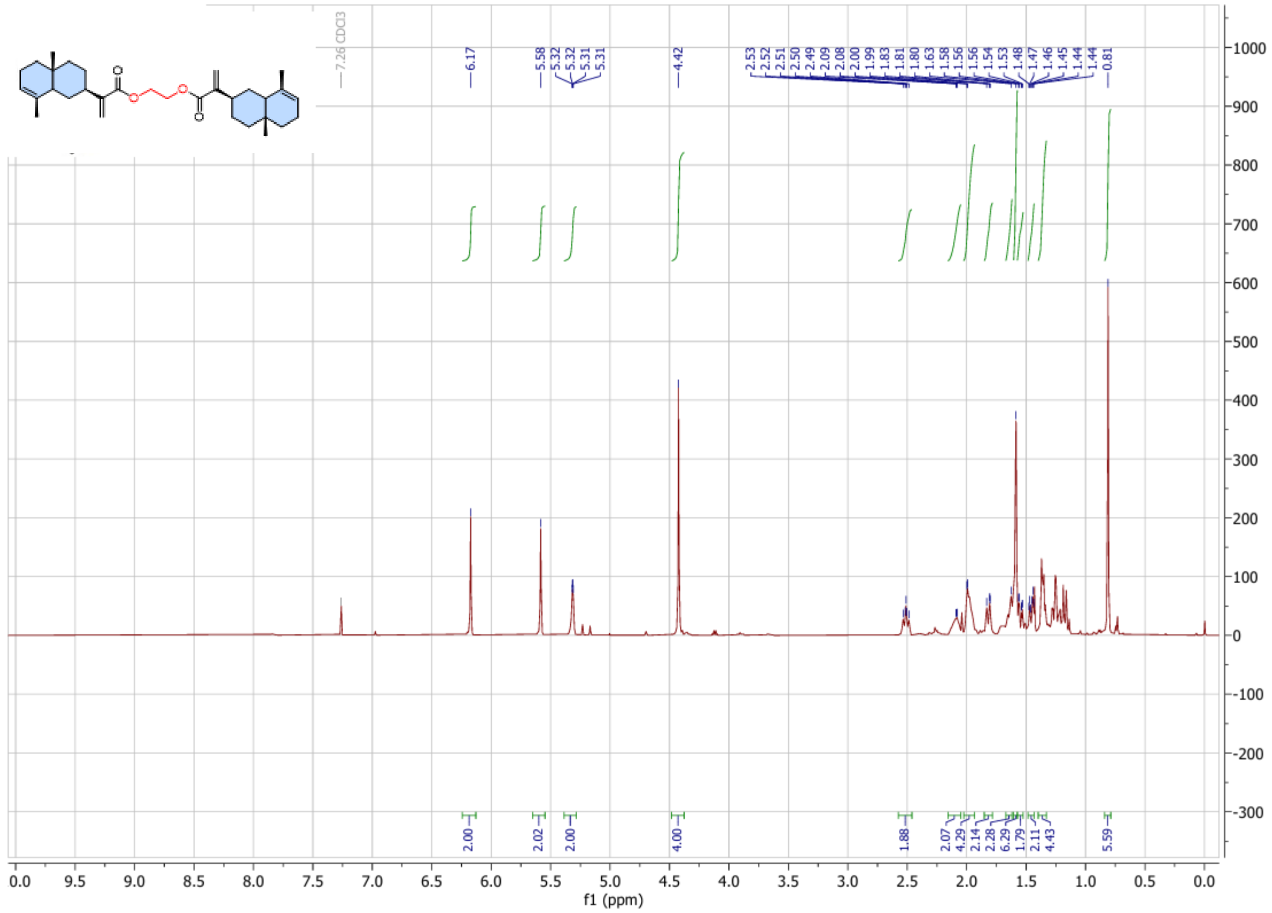

Figure S3. ^{1}H NMR (500 MHz, CDCl$_3$) spectrum of compound **6**.

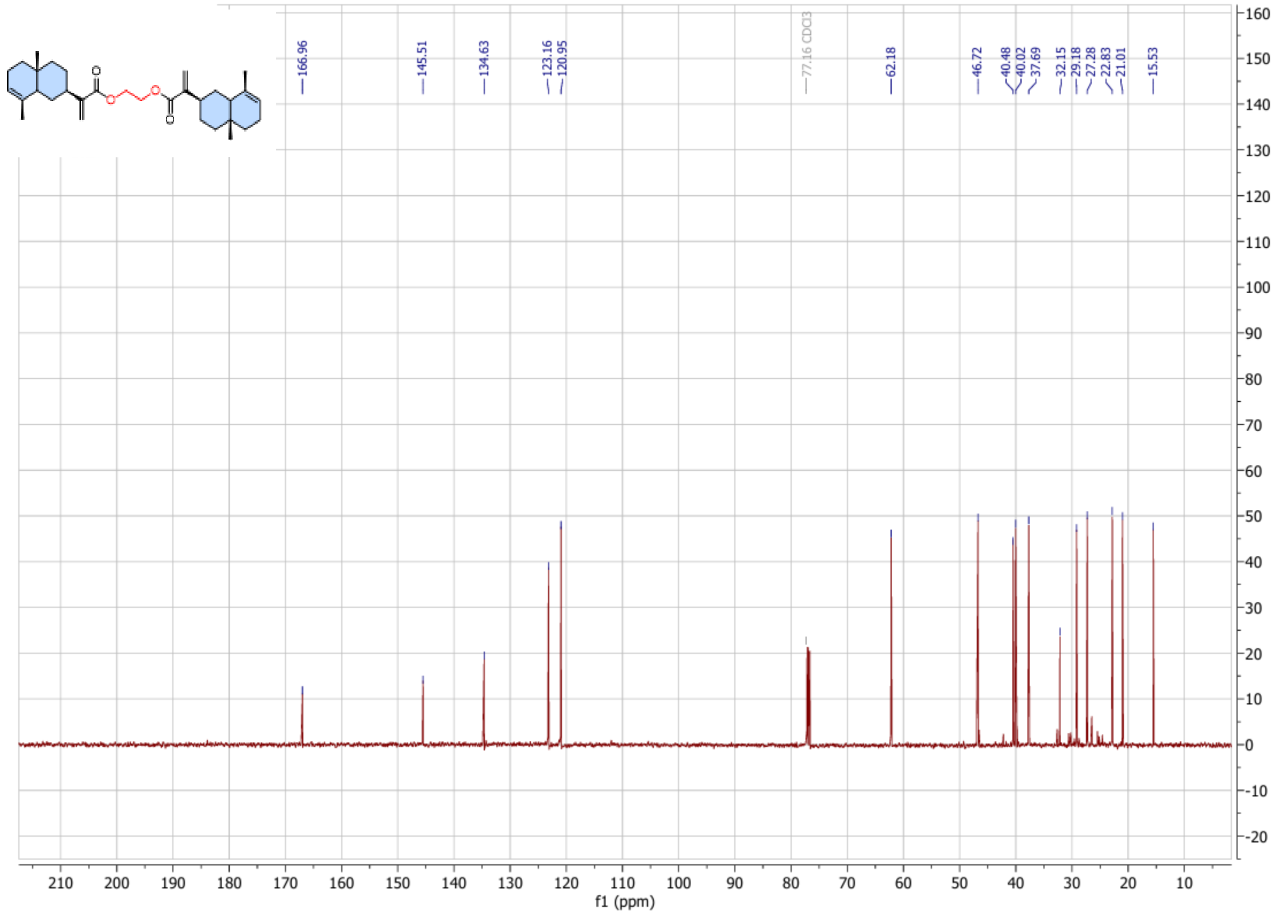

Figure S4. ^{13}C NMR (125 MHz, $CDCl_3$) spectrum of compound **6**.

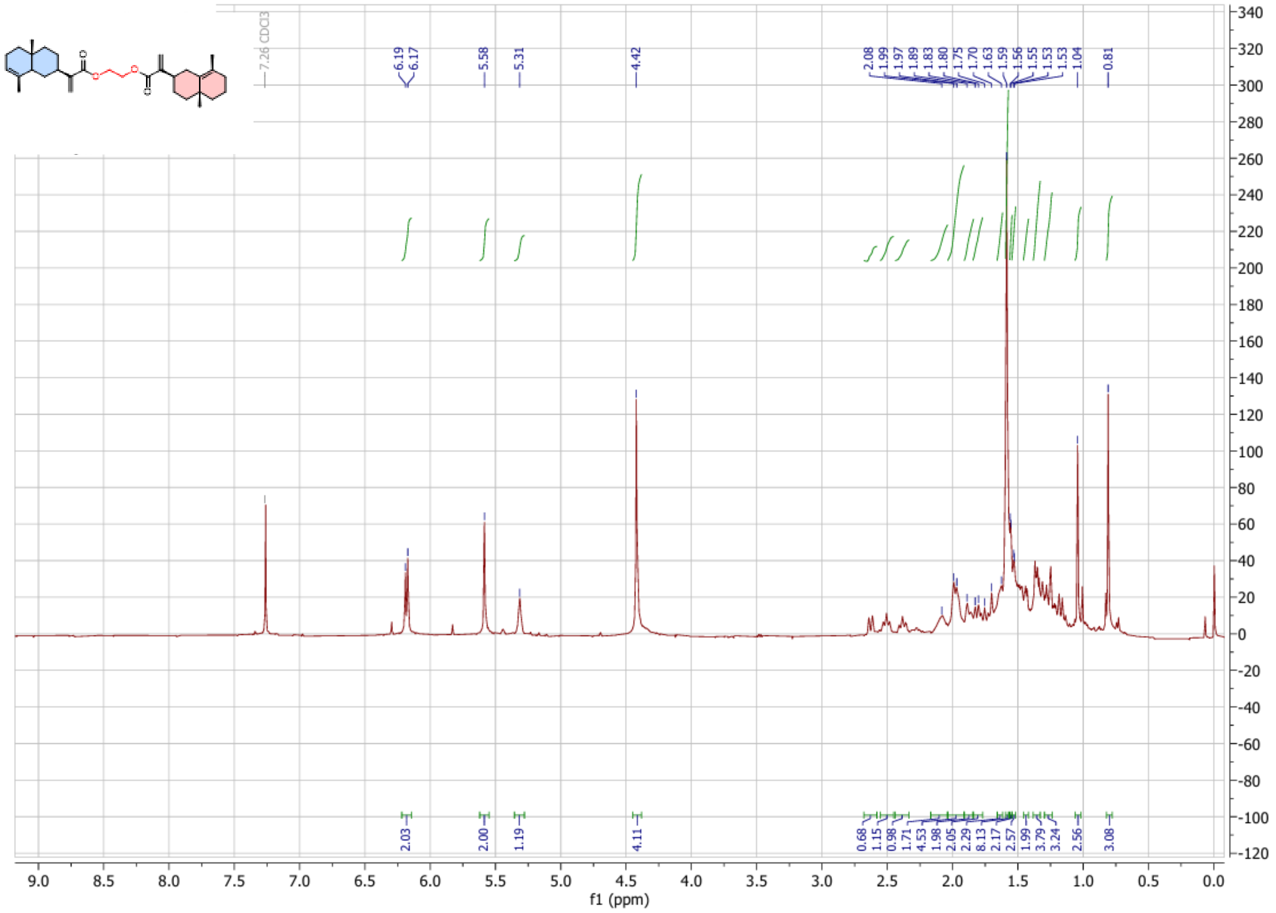

Figure S5. ^{1}H NMR (500 MHz, $CDCl_3$) spectrum of compound **7**.

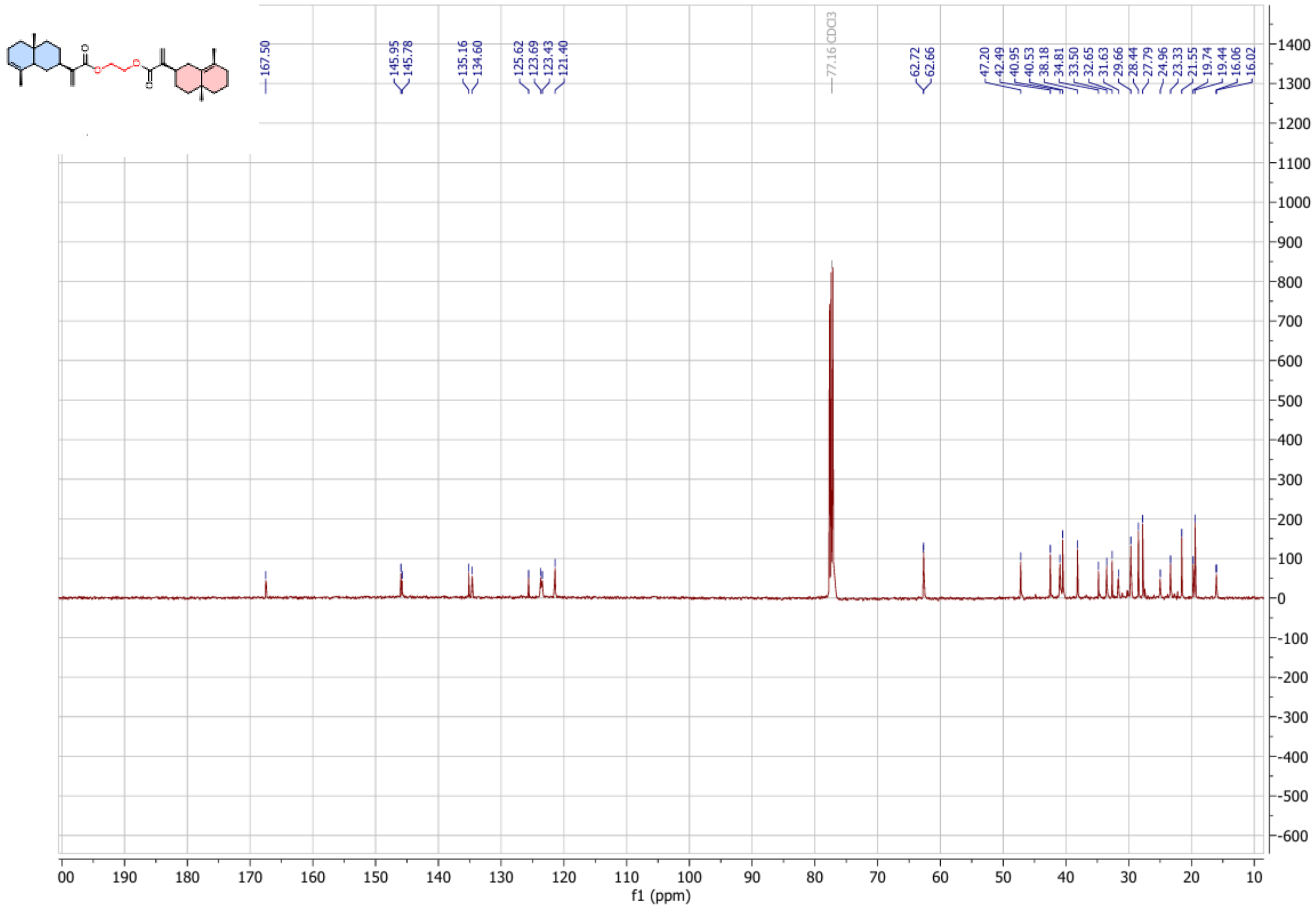

Figure S6. ^{13}C NMR (125 MHz, $CDCl_3$) spectrum of compound **7**.

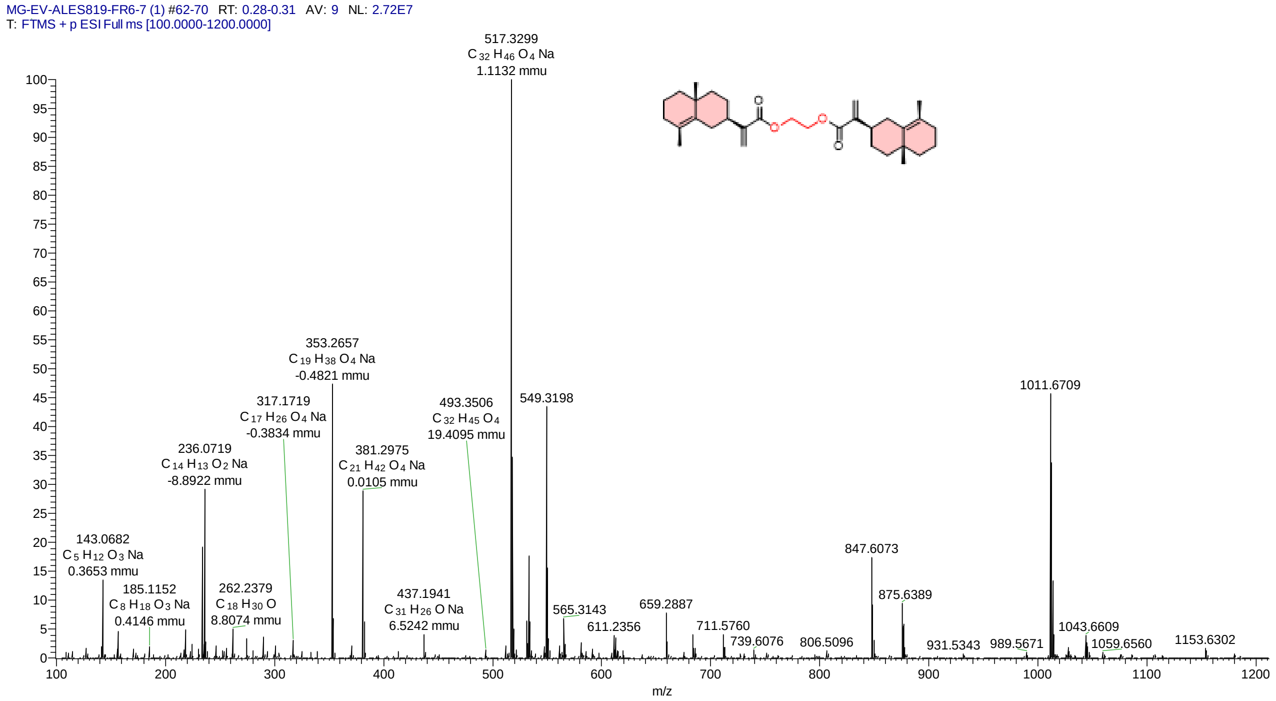

Figure S7. HR-ESIMS spectrum of compound **4**.

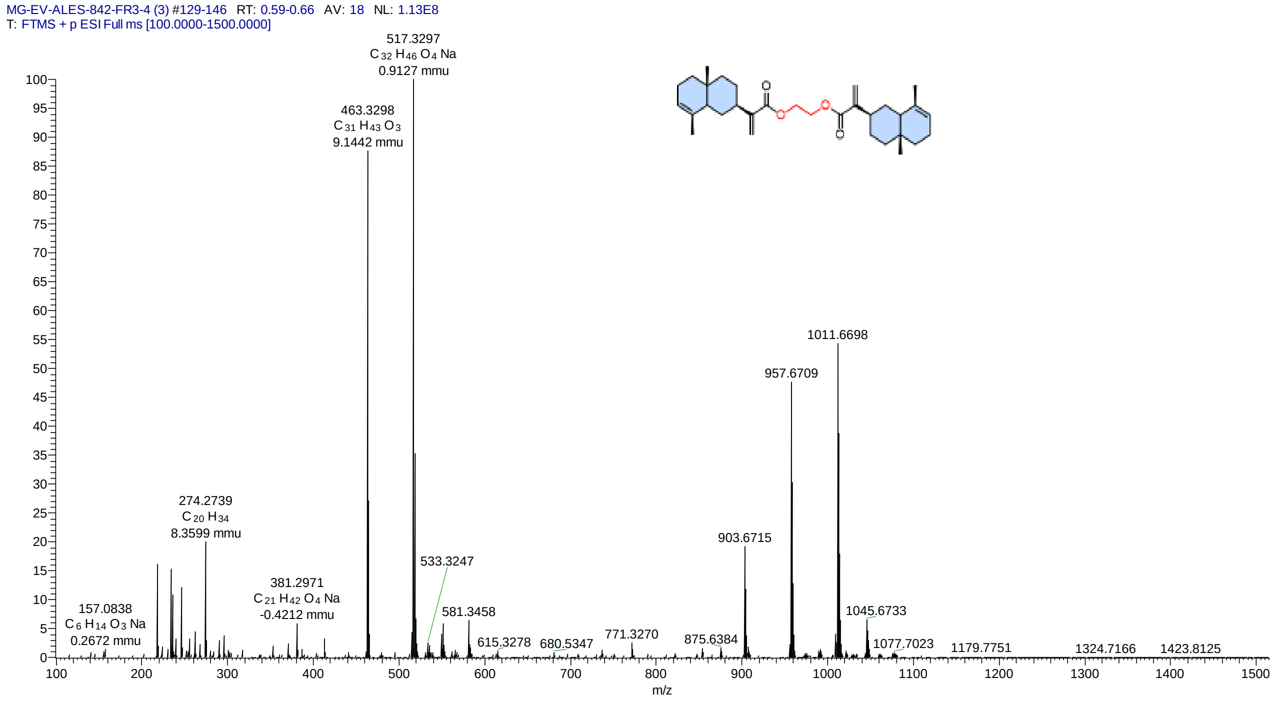

Figure S8. HR-ESIMS spectrum of compound **6**.

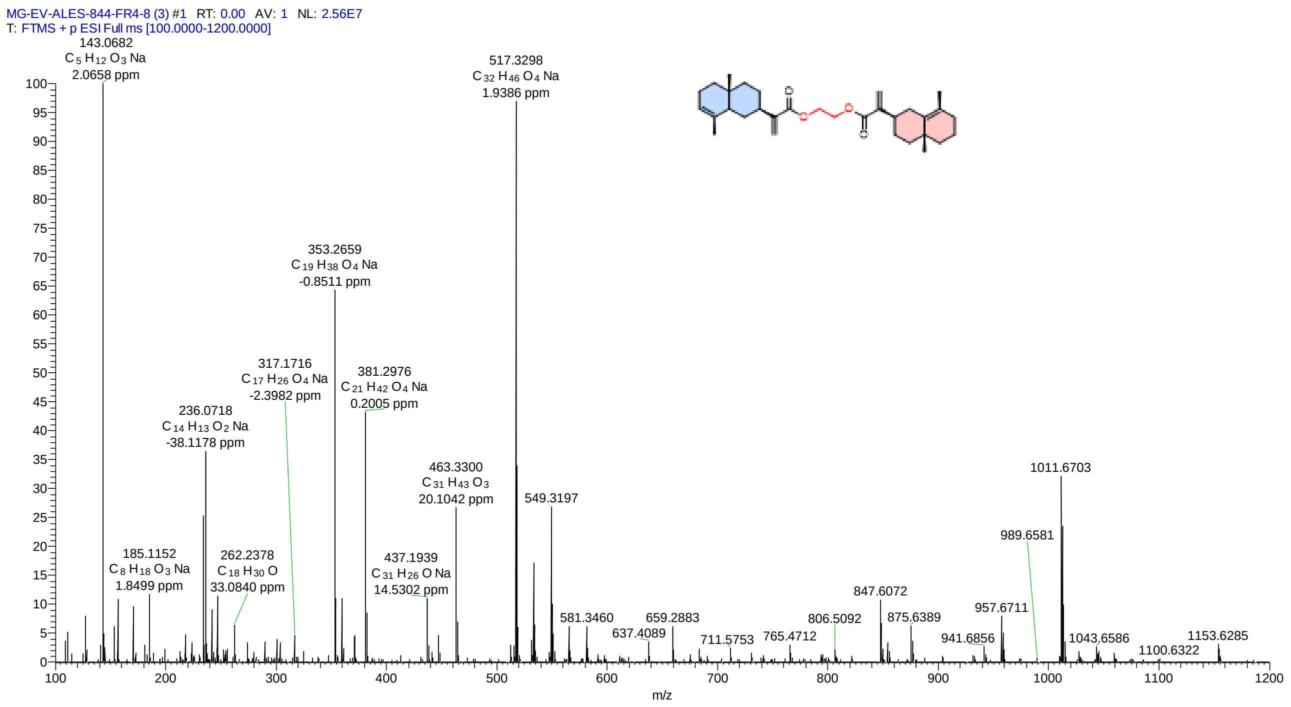

Figure S9. HR-ESIMS spectrum of compound **7**.
